# QSAR modelling of a large imbalanced aryl hydrocarbon activation dataset by rational and random sampling and screening of 80,086 REACH pre-registered and/or registered substances

**DOI:** 10.1371/journal.pone.0213848

**Published:** 2019-03-14

**Authors:** Kyrylo Klimenko, Sine A. Rosenberg, Marianne Dybdahl, Eva B. Wedebye, Nikolai G. Nikolov

**Affiliations:** Division of Diet, Disease Prevention and Toxicology, National Food Institute, Technical University of Denmark, Kongens Lyngby, Denmark; Chuo University, JAPAN

## Abstract

The Aryl hydrocarbon receptor (AhR) plays important roles in many normal and pathological physiological processes, including endocrine homeostasis, foetal development, cell cycle regulation, cellular oxidation/antioxidation, immune regulation, metabolism of endogenous and exogenous substances, and carcinogenesis. An experimental data set for human *in vitro* AhR activation comprising 324,858 substances, of which 1,982 were confirmed actives, was used to test an in-house-developed approach to rationally select Quantitative Structure-Activity Relationship (QSAR) training set substances from an unbalanced data set. In the first iteration, active and inactive substances were selected by random to make QSAR models. Then, more inactive substances were added to the training set in two further iterations based on incorrect or out-of-domain predictions to produce larger models. The resulting ‘rational’ model, comprising 832 actives and four times as many inactives, i.e. 3,328, was compared to a model with a training set of same size and proportion of inactives chosen entirely by random. Both models underwent robust cross-validation and external validation showing good statistical performance, with the rational model having external validation sensitivity of 85.1% and specificity of 97.1%, compared to the random model with sensitivity 89.1% and specificity 91.3%. Furthermore, we integrated the training sets for both models with the 93 external validation test set actives and 372 randomly selected inactives to make two final models. They also underwent external validations for specificity and cross-validations, which confirmed that good predictivity was maintained. All developed models were applied to predict 80,086 EU REACH substances. The rational and random final models had 63.1% and 56.9% coverage of the REACH set, respectively, and predicted 1,256 and 3,214 substances as actives. The final models as well as predictions for AhR activation for 650,000 substances will be published in the Danish (Q)SAR Database and can, for example, be used for priority setting, in read-across predictions and in weight-of-evidence assessments of chemicals.

## 1. Introduction

The aryl hydrocarbon receptor (AhR) is a ligand-dependent transcription factor that regulates the expression of genes, whose products are involved in multiple biological processes such as metabolism of endogenous and exogenous small molecules, as well as regulation of organ development and the immune system [1,2,3]. Due to its wide and important biological involvement, AhR continues to be a popular research area [4]. Some of the best characterized exogenous AhR ligands include dioxins, halogenated aromatic hydrocarbons (HAHs) and non-halogenated polycyclic aromatic hydrocarbons (PAHs). Further studies have identified a structurally diverse group of chemicals as activators of AhR [1]. Some of the genes regulated by AhR encode the enzymes P450 CYP1A1 and CYP1B1, involved in phase I metabolism, and sulfotransferase and UDP-glucuronosyltransferase, involved in phase II metabolism. Endogenous substances regulated by these enzymes include estrogen and thyroid hormones [5–10], and AhR can furthermore modulate the responsiveness of various hormone receptors by cross-talk [1]. One example of how interaction of man-made chemicals with AhR can interfere with normal physiology and lead to adverse health effects is given in an Adverse Outcome Pathway (AOP) under development that describes how the molecular initiating event (MIE) of AhR activation can upregulate thyroid hormone (TH) catabolism and lead to reduced TH levels resulting in adverse developmental outcomes [11]. Other AhR-related AOPs under development address the role of AhR activation for embryo or early life stage lethality [12, 13], liver dysfunctions [14, 15] and liver tumors [16]. Due to the involvement of AhR in toxic responses to chemicals [11], it is of high relevance to be able to identify and characterize chemical structures that activate AhR. A number of high-throughput screening (HTS) *in vitro* assays for AhR interaction have been developed and applied to screen thousands of small molecules [17,18]. Such data have previously been used in the development of quantitative structure-activity relationship (QSAR) models for AhR activation, e.g. QSAR models based on Tox21 HTS data under the Tox21 challenge in 2014 [17].

With the recent advances in *in vitro* assay technologies, data from HTSs for molecular and cellular responses are becoming more and more common in public databases such as the PubChem database [19– 21]. Such HTS datasets are often large, i.e. they can contain 100,000s of samples tested, and tend to be highly imbalanced towards a bigger class of inactives [20,22]. The availability of these large but highly imbalanced HTS experimental datasets has introduced new challenges when building global QSARs [20,22]. The datasets with 100,000s of entries are too large for most QSAR software to handle. Also, a very imbalanced distribution of actives to inactives poses a problem for many QSAR modelling algorithms because the modelling and validation will be biased toward correct prediction of the larger class [19]. One solution is, therefore, to select a subset to be used for QSAR training, e.g. with the aim of building models with good predictive performance and/or large applicability domain (AD) size. Suggestions on subset sampling and mining of large imbalanced HTS datasets have been published previously [20–23]. However, we are not aware of other studies reporting similar iterative QSAR training set selection approaches for sub-sampling of large experimental datasets in order to increase the applicability domain, as the one used in this study. The predictive performance of a QSAR, i.e. how good it is at making correct and reliable predictions, is strongly influenced by the quality of the structural and experimental data used for training [24,25]. For global QSARs, the size and balance of the training set, the distribution of training set structures in the chemical space, as well as the definition of the AD, also play a role in a model’s estimated predictive performance. Model coverage, defined as the AD size for a given set of substances, is the proportion of the set for which a QSAR model can make predictions with the reliability established in the QSAR validation. Increasing the training set size generally improves a model’s AD size as well as predictive performance, depending on the number and diversity of the added structures, the quality of the added experimental information, as well as the learning algorithm, see e.g. the discussion in [26].

The main aims of the present work were 1) to make QSAR models of high predictive performance using a highly imbalanced experimental data set from PubChem, and use these models; 2) to screen a set of 80,086 registered and/or pre-registered under the European Union chemicals regulation for Registration, Evaluation, Authorisation and Restriction of Chemicals (REACH) to identify potential AhR activators; and 3) to identify structural descriptors statistically most strongly associated with AhR activation and non-activation. To achieve these goals, another aim of equal importance was 4) to develop and test an approach to optimize training set selections from this imbalanced experimental data set. A large PubChem dataset with 324,858 chemical structures probing the classical AhR-gene activation mechanism in a *quantitative* HTS (*q*HTS) *in vitro* assay was curated and used to prepare training and test sets to build and validate global QSAR models. Due to the high ratio of 204,513 inactives to 925 actives in the curated dataset, we explored the effect of a stepwise expansion of the training set by rational selection of inactives on the predictive performances of the first four QSAR models and their coverage of 80,086 REACH substances. Furthermore, relevant data on luciferase inhibition, a potential artefact in the applied AhR activation assay, was taken into account to remove potentially false positive experimental results from the AhR activation dataset at the data curation step. To our knowledge, this step is also not reported in the existing published QSAR models for AhR. We have previously successfully modelled other xenobiotic receptor-related HTS data sets [27]. After the analysis of the impact of the rational selection approach, we added the external validation set actives and a random selection of the same number of inactives to the training sets and developed two final QSAR models which underwent cross-validation and external validation of specificity by a large set of inactives.

## 2. Materials and methods

### 2.1 Experimental datasets

A dataset consisting of structure information and *q*HTS *in vitro* data for human AhR activation and luciferase interference was used when constructing training and test sets. All data were downloaded from PubChem [21]. In total, 324,858 chemical substances have been tested in a primary singlicate screening for AhR activation (PubChem AID 2796), and given a PubChem activity score of 0–100 [18]. Of the 7,990 substances originally tested active in AID 2796, 2,281 have been retested in triplicate for AhR activation (AID 2845 [28]), and of these, 1,982 (86.9%) have been confirmed to be AhR activators, with a PubChem activity score of 10–100 [28]. The AhR activation *q*HTS *in vitro* assay applied in AID 2796 and AID 2845 is a luminescence-based assay using HepG2 cells stably transfected with AhR-dependent pGudLuc6.1-DRE plasmids [28]. Substances that activate AhR result in expression of the luciferase reporter gene, and the level of luciferase activity is an indirect measure of AhR activation [28]. Some substances can stabilize luciferase and increase its half-life resulting in its accumulation and a measured increase in luminescence signal [29], and such substances may be incorrectly interpreted as AhR activators in the applied AhR activation *q*HTS assay. We used experimental PubChem data from the luciferase inhibition/activation *q*HTS assay AID 5888342 [30] as a further counterscreen to identify such substances among the 1,982 confirmed AhR activators from AID 2845. We classified substances in AID 2845 with a PubChem activity score from 10 to 100 and a PubChem activity score of 0 in AID 588342 as active for AhR activation. Substances with a PubChem score of 0 in AID 2796 were classified as inactive for AhR activation. The remaining substances were classified as inconclusive for AhR activation.

### 2.2 Structure curation

**Fig 1:**
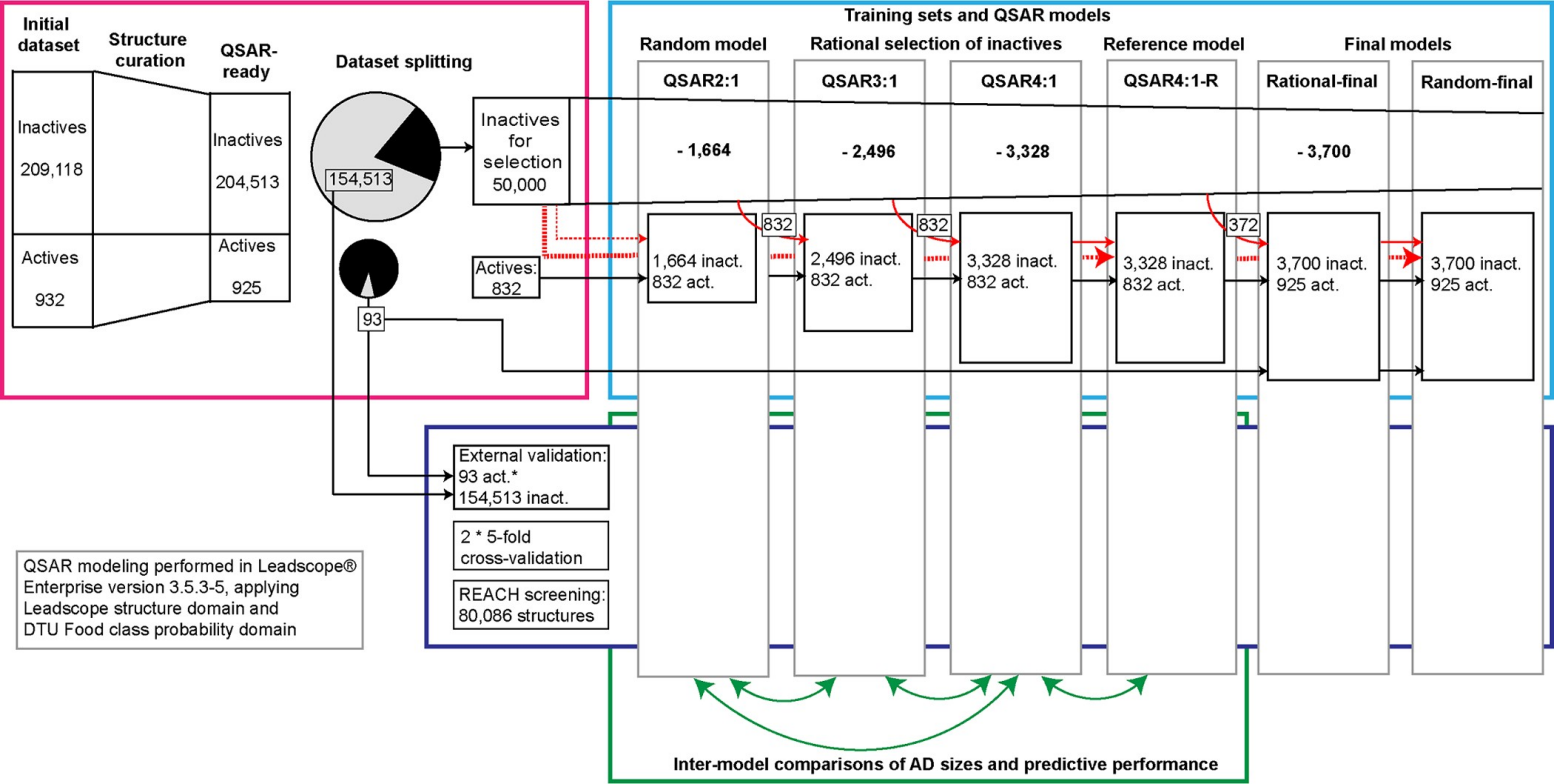
An overview of the workflow. Pink box: the steps of structure curation and preparation of test sets and datasets for training set construction. Light blue box: the steps of training set inactives selections and model building. Dark blue box: predicting the external validation test set, cross-validation sets and the REACH set in the four models. Green box: inter-model comparisons of the predictive performances from the external validations and the coverage of the REACH set.

The chemical structure curation was performed in OASIS Database Manager 1.7.3 [31] including algorithms developed in-house. Only structures exclusively containing atoms from the following list were kept: H, B, C, N, O, F, Na, Mg, Si, P, S, Cl, K, Ca, Br, and I. Records indicated by the processing software to contain errors in their structure information were removed from the dataset. Next, dissociation simulation was performed by breaking ionic bonds and the resulting structures were neutralized. After this, we removed substances containing two or more organic components (i.e. “mixtures”) and structures with less than two carbon atoms from the dataset. Canonized SMILES were generated for the remaining structures in the dataset. The numbers of QSAR-ready structures can be seen from Fig 1 (pink box).

*The two final QSAR models, Rational-final and Random-final were externally validated for specificity with the set of 154,513 inactives, but not for sensitivity as the 93 actives in the test set used for external validation of the previous models were included in the training sets of the two final models.

In the next step, identical QSAR-ready structures in the dataset were identified based on comparisons of the canonized SMILES strings. For identical structures with concordant experimental activities, only one instance of the structures was kept in the dataset, while if a group of identical structures had discrepant activities then the whole group was removed from the dataset. The final numbers of unique QSAR-ready structures can be seen from Fig 1 (pink box middle).

The initial dataset contained 932 actives and 209,118 inactives as defined in *2*.*1*. During the structure curation and duplicates handling steps described in *2*.*2*, a total of 4,612 structures were removed from the dataset, 2,909 due to the structural QSAR criteria and 1,703 due to structural duplicates, none of which had conflicting experimental results (Fig 1, pink box).

### 2.3 QSAR modelling approach

In this study, we used Leadscope Predictive Data Miner (LPDM), a component of Leadscope Enterprise version 3.5.3–5, to build QSAR models. It can handle organic chemical substances with a known and unambiguous 2D structure [32]. Briefly, upon dataset import LPDM calculates nine molecular descriptors (AlogP, Hydrogen Bond Acceptors and Donors, Lipinski Score, Molecular Weight, Parent Atom Number, Parent Molecular Weight, Polar Surface Area, Rotatable Bonds) and performs a systematic sub-structural analysis using a template library of more than 27,000 pre-defined structural keys for each chemical structure in the dataset [33]. For QSAR modelling in LPDM, the molecular descriptors and structural features are included in a preliminary descriptor set. From the preliminary descriptor set, an automatic descriptor pre-selection procedure in LPDM selects the top 30% descriptors according to Yates χ^*2*^-test for a binary response variable. For training sets with a binary response variable like in the present study, a partial logistic regression (PLR) predictive model is built starting with the pre-selected descriptors with further selection of descriptors in an iterative procedure, and selection of the optimum number of factors based on minimizing the predictive residual sum of squares. A description of the PLR method applied by the Leadscope system is available in [35] which also refers to other publications on the topic. Briefly, as described by Valerio et al. [35], *“PLR is used for a binary response variable and extracts factors by PLS using the responses as continuous data followed by logistic regression for classifications; this process is repeated until the criteria for optimum number of factors and features are reached*. *The binary classification model results are given as outcome probabilities from the logistic regression*.*”* LPDM has the option of building composite models, a type of ensemble models, for training sets with an imbalanced distribution of actives and inactives [34]. With this option a number of sub-models are created with a user-specified ratio of actives to inactives per sub-model training set. The positive prediction probability (see *2*.*4*) for a query chemical from a composite model is defined as the average of the positive prediction probabilities from all sub-models having the test chemical in their structural domain, if any [35] (otherwise the substance is out of AD). The models in LPDM were developed using three QSAR modelling approaches, which all underwent a 10 times 20%-out LPDM cross-validation:

1. A single model, i.e. a non-composite model using the full training set
2. A composite model, with sub-models of equal weight based on balanced training subsets
3. A composite ‘cocktail’ model, combining the single model from 1) with the sub-models of the composite model from 2) (this technique was first used by some of the authors in [36]).

### 2.4 Applicability domain definition

The definition of the AD applied in this study consists of two components: 1) the definition of a structural domain in LPDM, and 2) an in-house class probability refinement on the output from LPDM (to be denoted below as ‘DTU Food class probability refinement’):

1) For a query substance to be within LPDM’s structural domain it was required that it has at least 30% structural similarity using the Jaccard (also known as Tanimoto) coefficient [37] with a training set substance (based on Leadscope’s built-in fragment library), all molecular descriptors used in the model can be calculated and it contains at least one structural feature used in the model [35]. For test substances within the LPDM structural domain, a positive prediction probability, *p*, between 0 and 1, is given together with the prediction call; actives having a *p* ≥ 0.5 and inactives having a *p* < 0.5 [35].

2) The DTU Food class probability refinement served to exclude predictions that are likely to be less reliable, due to their positive prediction probability being close to the cutoff *p* = 0.5. For predictions to be within the AD we required a *p* ≥ 0.7 for active prediction calls (POS_IN) and a *p* ≤ 0.3 for inactive prediction calls (NEG_IN). Predictions within the LPDM structural domain but with an associated positive prediction probability in the interval 0.3 < *p* < 0.4 (NEG_OUT), 0.4 ≤ *p* < 0.6 (INC_OUT) and 0.5 ≤ *p <* 0.7 (POS_OUT) were defined as out of AD.

### 2.5. Random and rational training and test sets selections

After the curation step described in *2*.*2*, 925 active substances and 204,513 inactive substances were available for training set and test set selections. The subset of actives was split as follows: 93 (10%) and 832 (90%) out of the 925 AhR activators were randomly selected to be used in the test and training sets, respectively.

To find the maximal modelling capacity in our LPDM setting for the present dataset, we did a series of modelling experiments using training sets with different ratios of randomly selected inactives to actives. A training set with a ratio of 4:1 was the largest imbalanced training set that our installation of LPDM could efficiently model at the given time.

Two series of QSAR models were developed, based on rational and random selections of training set inactives, respectively. The goal of the rational selection of the training set inactives was to improve the prediction accuracy and the AD size. To pursue this, 50,000 randomly selected inactives from the total pool of inactives were used as a set to choose the inactive training set substances from. The remaining structures were included in the test set. A QSAR model with 832 actives and 1,664 inactives (to be called a ‘2:1 model’ as inactives were twice as many as actives) was developed. The inactives for this 2:1 model were selected randomly from the 50,000 inactives selection set. Three modelling approaches were used, as described in *2*.*3*, and the best performing modelling approach was selected based on the LPDM cross-validation results. The selected model was closed for further development and named QSAR2:1. Then the set of 50,000 minus the inactive structures in the 2:1 training set were predicted in QSAR2:1 (Fig 1, blue box). Based on these predictions, 832 new inactives were selected and added to the 2:1 training set to constitute a 3:1 training set as follows. The rational selection was done by selecting one fourth, corresponding to 208 structures, randomly from each of the four prediction outcome areas (defined in 2.4):

1. Out of LPDM structural domain
2. POS_OUT
3. NEG_OUT
4. POS_IN, i.e. here false positive (FP) predictions

The addition of structures from 1) through 3) mainly served to increase the chemical space of the subsequent training set with the purpose of increasing the overall AD size. The structures with POS_IN predictions, i.e. 4), were added with the purpose to improve the ability of the model algorithm to avoid deriving false activity features and thereby reduce the probability for false positive predictions. A similar but smaller effect on performance was expected from addition of the POS_OUT (2) and NEG_OUT selected structures.

The 3:1 training set was used for building a QSAR model using the selected modelling approach, and the model was closed and named QSAR3:1. The 50,000 minus the 3:1 training set inactive structures, i.e. 47,504 inactive structures, were then predicted in QSAR3:1 and based on these predictions, 832 additional inactives were selected following the same procedure as described above and added to the 3:1 training set to constitute a 4:1 training set (Fig 1, blue box). Again, the 4:1 training set was used for building a QSAR model using the selected modelling approach and the model was closed and named QSAR4:1.

In order to evaluate the effect of the rational selection steps, a reference model with a training set with a ratio of 4:1, i.e. consisting of 3,328 inactives randomly selected from the 50,000 selection set and the 832 actives, was made. This model was closed and named QSAR4:1-R.

In the end, final QSAR models were developed by adding the 93 external validation set actives and four times 93 inactives, i.e. 372 inactives, randomly selected from the selection set, to the 4:1 rational training set and to the 4:1 random training set, respectively. This gave two expanded 4:1 training sets, each with 925 actives and 3,700 inactives; i.e. 4,625 in total, which were applied to make two QSAR models using the cocktail approach, named Rational-final and Random-final.

### 2.6 Cross- and external validations of the QSAR models

The four models QSAR2:1, QSAR3:1, QSAR4:1 and QSAR4:1-R underwent a 10 times 20%-out cross-validation procedure in LPDM. The LPDM cross-validation is not an ‘independent’ cross-validation as the algorithm transfers knowledge from the full training set model to the smaller cross-validation models. Therefore, the LPDM cross-validation results were only used in a relative manner to guide the selection of the modelling approach (see *2*.*3*) and not to estimate absolute predictive performance.

To assess the predictive performances, the four models QSAR2:1. QSAR3:1. QSAR4:1 and QSAR4:1-R were subjected to two times five-fold cross-validations by an in-house procedure (to be denoted below as ‘DTU Food in-house cross-validation procedure’), where each generated sub-model was built without using any information outside its own training set. The four models also underwent an external validation using the test set of 93 AhR actives and 154,513 inactives (Fig 1, dark blue box). The two final QSAR models, Random-final and Rational-final underwent the DTU Food cross-validation procedure as well as external validation of specificity with the test set of 154,513 inactives. Cooper statistics (sensitivity—the percentage of experimental actives correctly predicted, specificity—the percentage of the experimental inactives correctly predicted, and balanced accuracy—the average of the sensitivity and specificity) was calculated for the test sets predictions within the defined AD. The predictive performances of QSAR4:1 and QSAR4:1-R were compared to assess the effect of the stepwise rational selection procedure (Fig 1, green box). The AD size for the test sets, i.e. the percentages of structures predicted to be within the defined AD, was also calculated for all four QSAR models.

### 2.7 Screening of 80,086 REACH substances for AhR activation

A collection of 80,086 REACH registered and/or pre-registered substances was extracted from the online Danish (Q)SAR Database structure set [38]. Of the 80,086 QSAR-ready REACH structures, 78,423 are REACH pre-registered and 10,046 are REACH registered (as of 7^th^ September 2018), i.e. there’s an overlap of 8,383 substances. The majority of the structures for the REACH pre-registered substances were originally curated from deliverable 3.4 of the OpenTox EU project [39]. All REACH structures were processed through the structure preparation steps described in *2*.*2*. The structures were screened through the QSAR2:1, QSAR3:1, QSAR4:1 and QSAR4:1-R AhR activation QSAR models for inter-model comparisons of coverage and predictive performance (Fig 1, dark blue box). Furthermore, the 80,086 REACH structures were screened through the two final QSAR models, Rational-final and Random-final, in order to obtain final predictions of activity. The proportion of the 80,086 QSAR-ready REACH structures predicted within the defined AD of each of the six QSAR models, respectively, as well as the activity distributions of the predictions were calculated.

To investigate the effect of the rational selection of inactives for the 4:1 training sets, an analysis of the AD size for the REACH set and the test sets for the models QSAR2:1, QSAR3:1, QSAR4:1 against the QSAR4:1-R model was performed (Fig 1, green box).

## 3. Results and discussion

In this study we developed QSAR models for AhR activation and explored how a large dataset with a high prevalence of inactives could be used for developing global QSAR models with improved AD sizes and predictive performances. We applied the developed models to screen 80,086 REACH substances for their potential AhR activation properties.

### 3.1 The datasets

The numbers of QSAR-ready structures and the distribution of active and inactive experimental results in the full curated dataset, the external validation test sets, inactives selection set, as well as the six training sets are summarized in Table 1.

**Table 1:** Overview of the datasets and their distributions of active and inactive experimental results.

Dataset overview	Actives	Inactives	Total
**Full dataset**	925	204,513	205,438
**Test set**	93	154,513	154,606
**Inactives selection set**	0	50,000	50,000
**2:1 training set (random)**	832	1,664	2,496
**3:1 training set (rational)**	832	2,496	3,328
**4:1 training set (rational)**	832	3,328	4,160
**4:1-R training set (random)**	832	3,328	4,160
**Rational-final training set (rational)**	925	3,700	4,625
**Random-final training set (random)**	925	3,700	4,625

### 3.2 Selection of the best model development approach

The 2:1 training set was used for building three types of QSAR models applying three different modelling approaches in LPDM (see *2*.*3*). Their LPDM cross-validation results are given in Table 2. These results were used for selecting the best modelling approach to be used for further modelling. As can be seen from Table 2, all three modelling approaches showed similar balanced accuracies in the 10 times 20%-out LPDM cross-validations: from 85.9% to 90.7%. The lower LPDM sensitivity of the single model was expected due to the imbalance of the training set towards the negatives. The 2:1 training set composite ‘cocktail’ models, approach 3, produced the highest numbers of true positive (TP) and true negative (TN) predictions while at the same time decreased the total number of false predictions compared to either of the two other approaches. Based on these numbers and also in order to achieve models with high specificity, i.e. producing fewer false positives without losing too much sensitivity, we selected the composite ‘cocktail’ modelling approach 3) for further modelling of the remaining training sets, 3:1, 4:1 and 4:1-R, as well as for developing the two final models.

**Table 2:** The results from the 10 times 20% out LPDM cross-validations of the three modelling approaches applied to the 2:1 training set (within the structural and probability AD).

	Predictions in LPDM and DTU Food domain						Statistical parameters		
Modelling approach	TP	TN	T_total_	FP	FN	F_total_	Sens., %	Spec., %	BA, %
**1. Single**	478	1,296	1,774	72	143	215	77.0	94.7	85.9
**2.Composite**	574	1,067	1,641	167	55	222	91.3	86.5	88.9
**3.‘Cocktail’**	544	1,264	1,808	104	68	172	88.9	92.4	90.7

TP = true positive, TN = true negative, T_total_ = total number of true predictions, FP = false positive, FN = false negative, F_total_ = total number of false predictions, Sens. = sensitivity, Spec. = specificity, BA = balanced accuracy. Sensitivity is defined as TP/(TP+FN). Specificity is defined as TN/(TN+FP). Balanced accuracy is defined as (sensitivity+specificity)/2.

### 3.3 Predictive performance assessment by cross-validation and external validation

After building the five models with the training set selections as described in *2*.*5*, they were all subjected to two times five-fold cross-validation (by DTU Food cross-validation procedure) and to external validation. The results are given in Tables 3 and 4, respectively.

**Table 3:** The results from the two times five-fold DTU Food cross-validation procedure of the cocktail models with different active-to-inactive ratios.

	Training set	QSAR2:1	QSAR3:1	QSAR4:1	QSAR4:1-R	Rational-final	Random-final
**Predictions in LPDM and DTU Food domain**	TP	982	724	648	994	775	1,132
	TN	2,247	2,58	3,079	4,657	3,679	5,228
	FP	263	423	513	432	552	486
	FN	204	248	281	188	264	178
	AD, %	74.0±1.7	59.7±2.4	54.3±2.1	75.4±1.2	57.0±1.4	75.9±1.1
**Cooper statistics**	Sens., %	82.8±3.4	74.5±4.3	69.6±5.5	84.0±3.8	74.5±4.7	86.4±2.6
	Spec., %	89.5±1.8	86.0±2.9	85.7±2.5	91.5±1.8	87.0±1.6	91.5±0.8
	BA, %	86.1±2.0	80.2±2.3	77.6±3.0	87.8±2.2	80.8±2.5	88.9±1.0
Prevalence of actives 1%	PPV, %	7.4	5.1	4.7	9.1	5.5	9.3
	NPV, %	99.8	99.7	99.6	99.8	99.7	99.9
Prevalence of actives 5%	PPV, %	29.3	21.9	20.4	34.2	23.2	34.9
	NPV, %	99.0	98.5	98.2	99.1	98.5	99.2
Prevalence of actives 10%	PPV, %	46.7	37.2	35.1	52.3	38.9	53.0
	NPV, %	97.9	96.8	96.2	98.1	96.8	98.4

TP = true positive, TN = true negative, T_total_ = total number of true predictions, FP = false positive, FN = false negative, F_total_ = total number of false predictions, Sens. = sensitivity, Spec. = specificity, BA = balanced accuracy, PPV = positive predictive value (the percentage of true positives among the predicted positives), NPV = negative predictive value (the percentage of true negatives among the predicted negatives)

**Table 4:** The results from the external validation of the models including model AD sizes for the test set.

		QSAR2:1	QSAR3:1	QSAR4:1	QSAR4:1R	Rational-final	Random-final
**Cooper statistics**	Sensitivity	87.5	84.6	85.1	89.1	N/A	N/A
	Specificity	90.1	95.4	97.1	91.3	96.8	91.3
	Balanced accuracy	88.8	90.0	91.1	90.2	N/A	N/A
**POS_IN**	TP	56	44	40	57	N/A	N/A
	FP	11,635	5,432	3,475	10,412	3,805	10,416
**NEG_IN**	TN	106,480	112,217	114,977	108,599	113,905	109,422
	FN	8	8	7	7	N/A	N/A
**Prevalence of actives 1%**	PPV, %	8.2	15.7	22.9	9.4	N/A	N/A
	NPV, %	99.9	99.8	99.8	99.9	N/A	N/A
**Prevalence of actives 5%**	PPV, %	30.9	48.2	59.7	34.1	N/A	N/A
	NPV, %	99.3	99.2	99.2	99.4	N/A	N/A
**Prevalence of actives 10%**	PPV, %	47.1	65.0	74.8	50.8	N/A	N/A
	NPV, %	99.5	99.2	99.3	99.7	N/A	N/A
**Coverage**	Of 93 actives	64	52	47	64	N/A	N/A
	(%)	(68.8)	(55.9)	(50.5)	(68.8)		
	Of 154,513 inactives	118,115	117,649	118,452	119,012	117,710	119,838
	(%)	(76.4)	(76.1)	(76.7)	(77.0)	(76.2)	(77.6)
	Total	118,179	117,701	118,499	119,076	N/A	N/A
	(%)	(76.4)	(76.1)	(76.6)	(77.0)	N/A	N/A

A decrease from 86.1% to 77.6% was seen in the cross-validation balanced accuracies from QSAR2:1 through QSAR3:1 to the QSAR4:1 model. According to the cross-validation results, the stepwise rational selection with addition of experimental inactives to the 2:1 and 3:1 training sets caused a decreased sensitivity, from 82.8% to 69.5%, as well as a decreased specificity, from 89.5% to 85.7%. Also, the coverage decreased from 74.0% to 54.3. These results were not surprising, given that 75% of the structures added to QSAR3:1 and QSAR4:1 from QSAR2:1 and QSAR3:1, respectively, i.e. prediction outcome areas 2–4 (POS_IN, POS_OUT and NEG_OUT, see *2*.*5*) were experimentally tested inactive but predicted to be associated with activity, i.e. contained positive structural alerts identified in the models. These types of structures were therefore “over-represented” in the left-out portions used to test the cross-validation sub-models as compared to the external validation test set, where they were not over-represented. This way, we have loaded the dice against the cross-validation results, particularly for specificity. Furthermore, as the number of true positives decreased and the number of false negatives increased from QSAR2:1 through QSAR3:1 to QSAR4:1, it is also clear that adding these many structures predicted to be associated with activity as experimental inactives has led to fewer and/or weaker positive alerts. This effect is discussed further with the external validation results below. Robustness of the models is slightly decreasing from the QSAR2:1, through the QSAR3:1 to the QSAR4:1 model with standard deviations increasing both for sensitivity and balanced accuracy. The QSAR4:1-R random model showed better robustness both for sensitivity, specificity and balanced accuracy than the QSAR4:1 rational model.

Each of the two final models, Random-final and Rational-final, showed similar or better sensitivity, specificity and balanced accuracy than their respective predecessors by cross-validation. Both models also showed better robustness (lower standard deviations) than their predecessor, with the Random-final model showing the best robustness of all models developed in this study. Like for their predecessors, the Random-final model showed better coverage of the AhR activators than the Rational-final model, in total around 26% (Rational-final TP+FN = 1,039 and Random-final TP+FN = 1,310). The slightly worse results for the rational models compared to the random models may both reflect that some of the positive alerts were disturbed by the deliberate addition of experimentally inactives with POS_IN, POS_OUT and NEG_OUT predictions as well as be related to the loading of the dice against good cross-validation results by adding these structures that are difficult for the models to predict correctly.

Positive (PPV) and negative (NPV) predictive values were calculated for all six models under three different assumptions about the prevalence of actives in the tested chemical universe: 1%, 5% and 10%. The PPV values for each model increase considerably with the increased prevalence of actives, while NPV is almost unchanged. Except for the experimental training set, the actual prevalence of actives in different chemical universes is to our knowledge unknown. As discussed above, the cross-validation results for the rational models are hampered by the training sets containing hard-to-predict inactive experimental results, which is also reflected in the PPV and NPV values.

A small increase from 88.8% to 91.1% was seen in the external validation balanced accuracies from QSAR2:1 through QSAR3:1 to the QSAR4:1 model. The stepwise rational selection with addition of experimental inactives to the 2:1 and 3:1 training sets gave a total increase in specificity of 7%, i.e. from 90.1% in QSAR2:1 to 97.1% in QSAR4:1. This increase in specificity had a dramatic effect on the number of false positives, which was reduced to one third from 10,412 in the QSAR4:1-R to 3,475 in the QSAR4:1 model. As the AhR full data set had 932 confirmed actives and 209,118 inactives there is a prevalence of inactives of 99.6%, meaning that only 0.4% of this chemical universe tested active in the assay. For an effect in a universe highly skewed towards inactives it is very important to have a high-specificity model to be able to achieve a positive predictive value (true positives out of total predicted positives) not approaching zero.

The sensitivity was more or less unaffected and ranged from 84.6% to 87.5% without a clear trend between the models, and these small differences in the sensitivities are possibly mainly due to noise from applying the rather small test set of 93 actives. However, the coverage of the actives went down from 68.8% to 50.5% meaning that in effect a lower number of actives were predicted by the QSAR4:1 rational model. The smaller coverage of the actives must have been due to the DTU Food class probability part of the AD definition rather than to the LPDM’s structural domain part of the AD definition as the structures were covered by the 2:1 model and addition of more structures would not make the structural domain smaller. So the declining coverage of the actives must be due to the rational models containing fewer and/or weaker positive alerts than the random models, resulting in some of the actives in the external validation set being rendered out of domain due to the positive prediction probability moving down into the ‘grey zone’ between 0.3 and 0.7. This shows that the adding of POS_IN and POS_OUT predictions, possibly also to some extent NEG_OUT predictions, to produce the 3:1 and 4:1 training sets not only led to increased specificities, as some assumed incorrect positive alerts were eliminated, but it apparently also disturbed some of the correct positive alerts. Possible theoretical explanations for this effect could be modelling limitations to precisely discriminate between actives and very structurally similar inactives, and another one could be that some of these added substances were actually false negatives by the experimental test. We have not found measures of the intra-laboratory and/or inter-laboratory reproducibility of the experimental test or comparisons to other experimental tests for AhR activity in the scientific literature, so we cannot compare to an estimated ‘sensitivity’ of the experimental test used for the data set applied in this study.

Positive (PPV) and negative (NPV) predictive values were calculated for the four initial models under three different assumptions about the prevalence of actives in the tested chemical universe: 1%, 5% and 10%. The PPV values for each model increase considerably with the increased prevalence of actives, while NPV is almost unchanged. Furthermore, for each of the prevalence assumptions the PPV for the rational QSAR4:1 model is clearly highest due to this model having the highest specificity. Especially in case of a very skewed universe with only few active substances, high specificity is important for the trustworthiness of an active prediction.

The coverage of inactives remained more or less unchanged between the four models. The iterative addition of experimentally negative training set substances which were predicted out of LPDM structural domain was expected to generally increase the model AD size, i.e. also coverage of inactives. Addition of experimentally negative training set substances which were predicted negative or positive but out of the DTU Food class probability refinement domain, or positive inside the DTU Food class probability refinement domain, was expected to refine the positive and negative alerts in the next iteration model resulting in these types of substances achieving lower positive prediction probabilities. This could lead to these types of substances either being predicted negative inside the DTU Food class probability refinement domain, or being predicted to be in the DTU Food class probability refinement domain ‘grey zone’ between 0.3 and 0.7, i.e. possibly leading to a maintained or decreased domain.

The test set consisted of 93 actives (for models QSAR2:1, QSAR3:1, QSAR4:1 and QSAR4:1-R) and 154,513 inactives (for all models). TP = true positive predictions, TN = true negative predictions, FP = false positive predictions, FN = false negative predictions, PPV = positive predictive value, NPV = negative predictive value.

A comparison of the external validation statistical parameters between QSAR4:1 and QSAR4:1-R models showed that the QSAR4:1 model had a higher specificity and lower sensitivity compared to QSAR4:1-R random model (Table 4). The positive effect on the specificity was an expected result from the procedure of rational addition of inactives selected among the POS_IN, POS_OUT and to some extent NEG_OUT predictions produced by the preceding models. Inclusion of these structures with experimentally tested false positive predictions in the training set can help the subsequent model train on a chemical space which balances better the positive and negative evidence for certain chemical classes. Even though the increase in the training set size led to an increase in balanced accuracy and specificity, surprisingly it did not result in a wider AD neither for the QSAR4:1 nor the QSAR4:1-R model. Another shortcoming of the QSAR4:1 model is the lower coverage of the external test set positive substances (50.5%) compared to the other models, although the coverage percentages of positives have less significance due to the small number of positives in the test set.

The Rational-final model and the Random-final model were assessed by external validation for specificity only since the external validation set only contained inactives. Both models according to the external validation with 154,513 inactives showed similar specificities to their respective predecessors, with 96.8% for the Rational-final model and 91.3% for the Random-final model.

Furthermore, the two final models underwent a Y-randomization test to investigate the risk of chance correlations in the models. In two separate exercises for the Rational-final and the Random-final model, respectively, each substance in the training set was assigned activity or inactivity by random, in total 2,313 were assigned activity and 2,312 inactivity. QSAR models were developed on the Y-randomized training sets and subjected to two times five-fold cross-validation by the DTU Food cross-validation procedure. For the Rational-final model, sensitivity was 51.1±3.7% and specificity was 48.4±3.7%. For the Random-final model sensitivity was 49.7±4.1 and specificity was 48.2±4.9%. The results clearly show that the randomized models have no predictive value, leaving no doubt that the risk of chance correlation for the Rational-final and the Random-final models is practically non-existent.

### 3.4 Top structural features in the Rational-final and Random-final models

**Fig 2:**
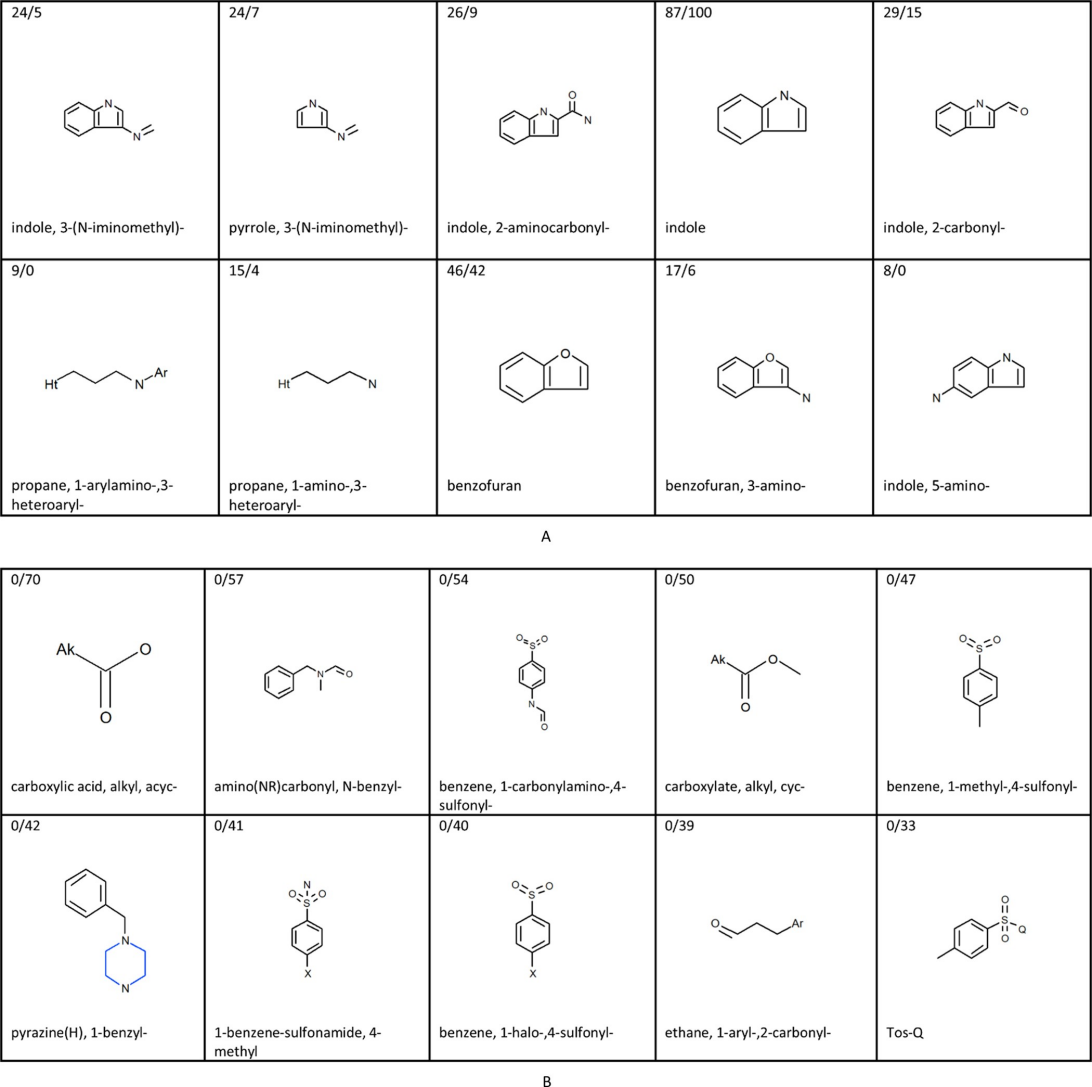
The most significant activity and inactivity structural features occurring in the Rational-final model. (A) Structural features alerting for activity in the Rational-final model. (B) Structural features alerting for inactivity in the Rational-final model. The selection of activity features was based on a ranking by the formula |0.2 - $\bar{x}$|∙ χ^2^, where $\bar{x}$ is the mean activity of all training set structures containing the feature. The selection of inactivity features was done by significance (χ^2^) among the ‘pure’ inactivity features, i.e. only appearing in inactive substances. In both cases χ^2^ denotes Chi-square independence test with one degree of freedom with Yates’ correction.

**Fig 3:**
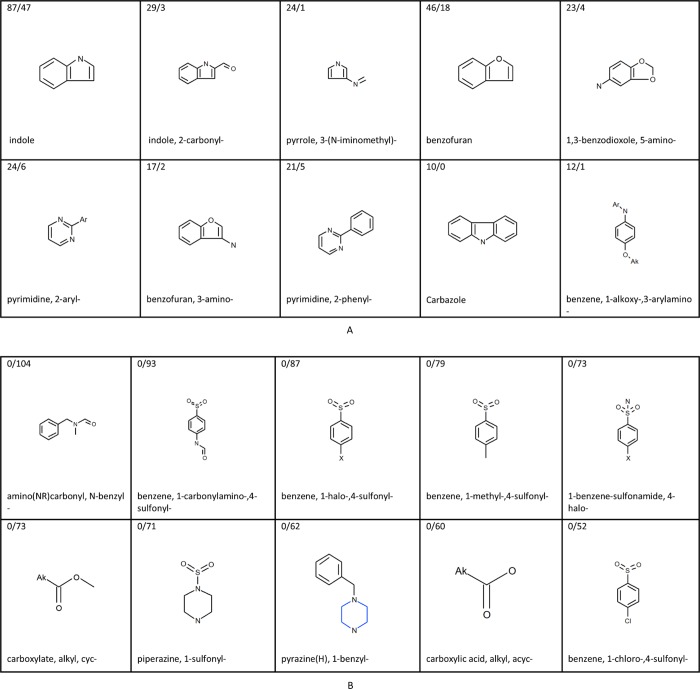
The most significant activity and inactivity structural features occurring in the Random-final model. (A) Structural features alerting for activity in the Random-final model. (B) Structural features alerting for inactivity in the Random-final model. The selection of activity features was based on a ranking by the formula |0.2 - $\bar{x}$|∙ χ^2^, where $\bar{x}$ is the mean activity of all training set structures containing the feature. The selection of inactivity features was done by significance (χ^2^) among the ‘pure’ inactivity features, i.e. only appearing in inactive substances. In both cases χ^2^ denotes Chi-square independence test with one degree of freedom with Yates’ correction.

The most significant and discriminating predictive structural features associated with actives and inactives in the Rational-final model and the Random-final model, respectively, are shown in Fig 2 and Fig 3. The highest ranking structural features associated with activity included for both models in the majority of cases heteroarenes such as derivatives of indole, pyrrole, benzofuran and pyrimidine. Four of the top-10 structural features for activity are identical between the two models, i.e. indole, 2-carbonyl indole, 3-(N-iminomethyl) pyrrole and 3-aminobenzofuran. Fig 2 and Fig 3 confirm the earlier reported high structural diversity of AhR activators and for example presence of heteroarenes in some activators, see e.g. [1], although halogenated or polycyclic aromatic hydrocarbon activity features are not present in Fig 2 or Fig 3. Structural features containing halogen are, however, present in the full lists of structural features for activity for both models, but with lower ranking. For both models also goes, that their full lists of structural activity features contains structural features with a naphthalene moiety, but none contain a bigger polyaromatic part. Benzo(e)pyrene and phenanthrene were tested inactive in the PubChem experimental data set, and dioxin was not tested in the set. Furthermore, a number of continuous descriptors were present in the top-40 descriptors for the two models, i.e. molecular weight, number of rotatable bonds and polar surface, as well as hydrogen bond acceptors for the Rational-final model and hydrogen bond donors for the Random-final model. It would have been interesting to observe if pKa would have come out among the top continuous descriptors had it been included in the descriptor pool for the modeling. This might theoretically have improved the models, since ionization state of molecules may influence receptor activation. The top structural features associated with inactivity in the two models included derivatives of sulfonyl, sulfonamide, carboxylic acid, carboxylate, and pyrazine. Seven of the top-10 structural features for inactivity are identical between the two models. The top inactivity structural features in the Rational-final model were present in fewer substances than the top inactivity structural features in the Random-final model, because the training set for the Rational-final model had deliberately been fed with substances for which positive activity had to some extent been predicted, thereby indirectly leading to feeding fewer training set substances with strong inactivity features.

The activity fragments are ranked by the score |0.2 - $\bar{x}$|∙ χ^2^, where $\bar{x}$ is the mean activity of all training set structures containing the feature and χ^2^ denotes Chi-square independence test with one degree of freedom with Yates’ correction. The intention of this score was to take into account both the discriminative power (how well a fragment discriminates between the active and the inactive class) and the significance of the fragment. The discriminative power is expressed by the difference between the active/inactive ratio of the training set substances containing the fragment and 0.2 (the active/inactive ratio of the whole training set): the higher this difference, the more the fragment is over-represented in one of the classes. The selection of inactivity features was done by significance (χ2) among the ‘pure’ inactivity features, i.e. only appearing in inactive substances.

In the training sets of the two final models where the ratio of inactives to actives is 4:1, a feature not associated with activity would on the average occur in four times as many inactive training set substances than active ones, and therefore 0.2 was chosen as the proportion of actives in the training set. ‘Ak’ matches saturated carbon. ‘Ar’ matches aryl. Numbers in the upper left corners display the number of AhR activators over the inactives in the Rational-final training set containing the specific structural feature. For example, the first structural feature alerting for activity in the Rational-final model, 3-(N-iminomethyl)-indole, was present in 24 AhR activators and 5 AhR inactives in the model training set. The ratio of inactives to actives of 4:1 also explains why an alert can be positive even though it occurs in fewer active than inactive training set substances: if the proportion of actives among the structures the alert occurs in is significantly more than 20%, it may be associated with activity.

### 3.5 REACH screening results

**Fig 4:**
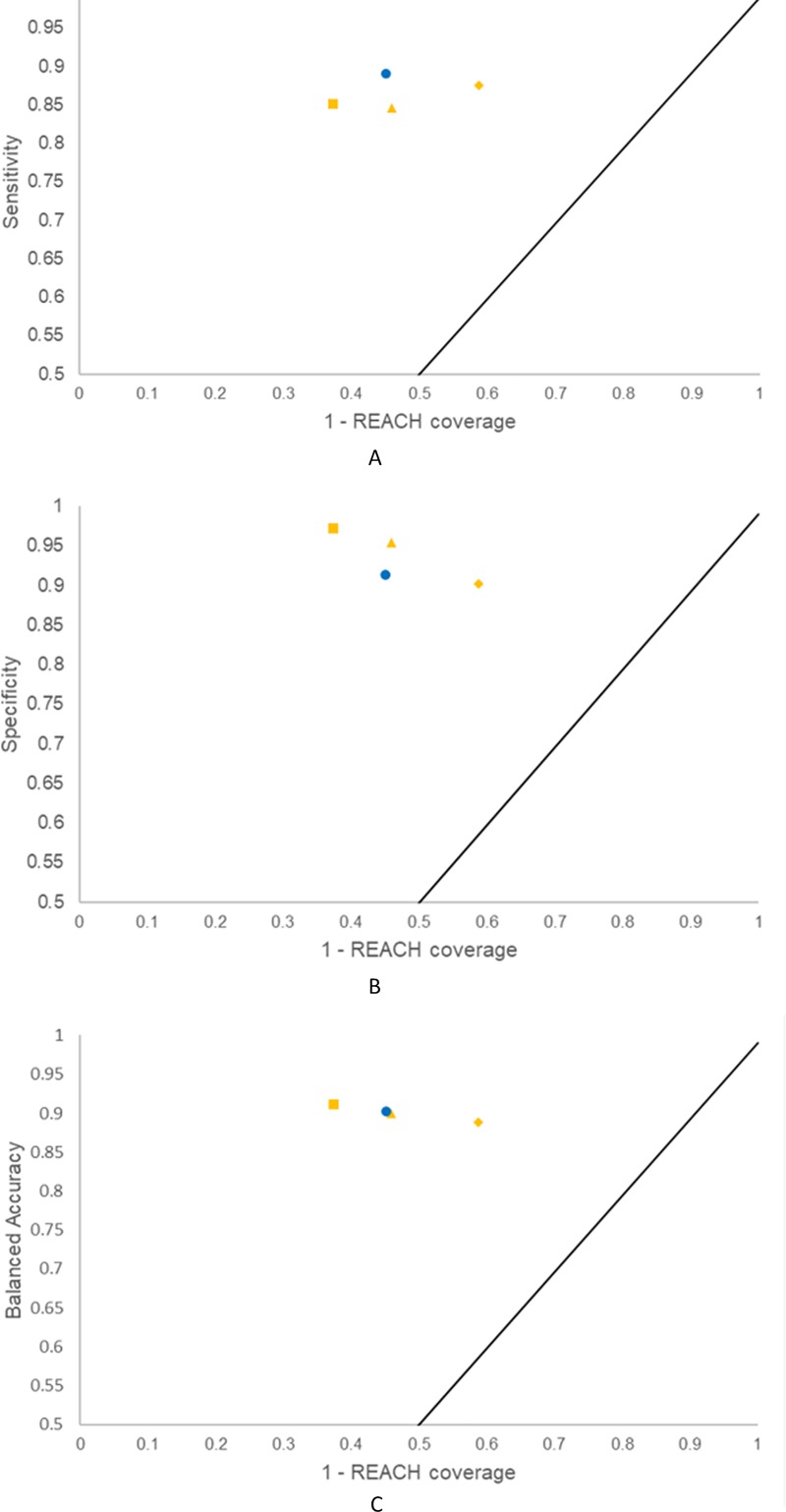
Performance of QSAR2:1, QSAR3:1, QSAR4:1 and QSAR4:1R vs. REACH coverage. The performance is described by Sensitivity (A), Specificity (B) and Balanced Accuracy (C). The following tokens correspond to the rational selection approach: a yellow diamond for QSAR2:1, a yellow triangle for QSAR3:1 and a yellow square for QSAR4:1. The blue circle corresponds to the random selection approach for QSAR4:1-R.

Another focus of this study was to make predictions for the REACH set of 80,086 substances, and specifically to explore how the selection of inactives for the training sets would affect the model AD sizes for this set. In Table 5, the results for the six models are given, and as can be seen all models showed coverages from 41.2% to 63.1%. Fig 4 contains performance plots on the model AD sizes for the REACH set.

**Table 5:** Number of substances covered, (% of screened REACH substances), number of predicted actives (% of covered) and number of predicted inactives (% of covered) from predicting the REACH set of 80,086 substances.

	Coverage (%)	POS_IN (%)	NEG_IN (%)
**QSAR2:1**	33,013 (41.2)	2,842 (8.6)	30,171 (91.4)
**QSAR3:1**	43,328 (54.1)	1,659 (3.8)	41,669 (96.2)
**QSAR4:1**	50,136 (62.6)	1,477 (2.9)	48,659 (97.1)
**QSAR4:1- R**	43,994 (54.9)	2,952 (6.7)	41,042 (93.3)
**Rational-final**	50,561 (63.1)	1,256 (2.5)	49,305 (97.5)
**Random-final**	45,557 (56.9)	3,214 (7.1)	42,343 (92.9)

There was a consistent increase in the coverage of the REACH set with the increase of the training set size. This was an expected effect of the gradual increase in training set size, and was an effect of the large increase in NEG_IN predictions, which was bigger than the decrease in the POS_IN predictions (Table 5). Also, the QSAR4:1 model made by means of the rational training set selection has better coverage than the corresponding QSAR4:1-R random model. Despite the same number of actives and inactives in the QSAR4:1 and QSAR4:1-R training sets, the coverage of the REACH set goes up from 54.9% in QSAR4:1-R to 62.6% in QSAR4:1, i.e. almost 15% larger. This is most likely an effect of the rational selection steps, even though this enlargement of the AD size was not seen for the external validation. For the REACH set, QSAR4:1 produced more NEG_IN predictions, i.e. 48,659 versus 41,042, with a smaller absolute decrease in its number of POS_IN outputs, i.e. 1,477 in QSAR4:1 versus 2,952 in QSAR4:1-R.

The increased number of NEG_IN predictions produced by QSAR4:1 compared to QSAR4:1-R is likely a result of an increased structural diversity of inactives in the rational selected training set. This increase in structural diversity and the AD was mainly driven by the addition of structures with predictions out of LPDM structural domain.

Adding structures with NEG_OUT predictions may have helped the subsequent model to make more reliable predictions, i.e. NEG_IN, for these types of structures. The addition of inactive structures with false POS_IN and POS_OUT predictions in the intermediate models has likely helped the QSAR4:1 model reduce its rate of FP predictions, and is part of the reason for the smaller number of POS_IN REACH predictions generated from QSAR4:1. However, since the rational addition of structures was only aimed at increasing the number and diversity of inactive structures in the training set without a corresponding increase in training set actives, the addition of structures in the POS_IN and POS_OUT prediction areas has also resulted in a certain loss of TP predictions produced by QSAR4:1. This can also be seen in the results from the test set, where QSAR4:1 resulted in 40 TP predictions out of the 93 test set actives as opposed to the 53 TP predictions from QSAR4:1-R (Table 3).

The Rational-final model predicted 1,256 substances (2.5% out of the 50,561 substances in AD) to be POS_IN versus the Random-final model, where 3,214 (7.1% out of the 45,557 substances in AD) were predicted POS_IN. Of the 80,086 REACH substances, 39,478 were inside AD of both models. Of these, 1,135 substances were predicted positive in both models and 38,305 were predicted negative in both models. The two models only disagreed for 38 substances: 4 were predicted positive in the Rational-final model and negative in the Random-final model, and 34 were predicted positive in the Random-final model and negative in the Rational-final model. This results in a Matthews correlation coefficient (MCC) of 0.983.

### 3.6 Availability of results

All data used in this study are freely available from a public repository [18]. In addition, we make the training sets of all six QSAR models developed in this study as well as the external validation sets (93 actives and 154,513 inactives) available online at http://qsar.food.dtu.dk/download/ahr/training_sets_DK_QSAR_DB_AhR.zip and http://qsar.food.dtu.dk/download/ahr/test_sets_DK_QSAR_DB_AhR.zip, respectively, including chemical structure and AhR activity. Predictions for 650,000 structures from the Rational-final and Random-final models, including the 80,086 REACH structures, will be made freely available from the online Danish (Q)SAR Database [38]. The two models will also be made available for prediction of user-submitted structures in a coming free online Danish (Q)SAR Models sister-site to the Danish (Q)SAR database at the DTU homepage.

## 4. Conclusions

The main aims of the present work were to make QSAR models of high predictive performance and in the process develop and test an approach to optimize training set selections, identify structural descriptors associated with AhR activation and non-activation, and screen a set of 80,086 REACH substances to identify potential AhR activators.

Regarding the developed rational training set selection approach, we conclude that the stepwise rational selection of training set inactive structures from a very large and imbalanced dataset improved model specificity and overall coverage compared to random selection, however at the cost of sensitivity. The best performance for sensitivity and coverage of actives was obtained by a random selection approach. The statistical performance of the developed models was determined by two times fivefold cross-validations and external validations with an external experimental validation set with 93 actives 154,513 inactives. Results from the cross-validations showed that both sensitivities and specificities were lower for the rational models than for the QSAR4:1-R random model, in fact performance and robustness decreased by each consecutive iteration of the rational selection approach. However, the large external validations showed that the rational models had higher specificity than the random model. Since more robust results for specificity performance can be achieved with external validation applying such huge test set, combined with the presented interpretation of the poor cross-validation results for the rational models as a kind of artefact to the rational selection process, more weight is given to the external validation result. According to the external validation, the specificity improved from 91.3% for the QSAR4:1-R random model to 97.1% for the QSAR4:1 rational model. For a rare activity like AhR activation with only 0.4% tested active out of more than 200,000 substances experimentally tested, this specificity improvement as a result of the rational selection of training set inactives is important to decrease the number of false positives, thereby increasing confidence in positive predictions. According to the external validations, the sensitivity was decreased from 89.1% for the QSAR4:1-R random model to 85.1% for the QSAR4:1 rational model. Overall, the external validations showed that the two models had high predictive performances with balanced accuracies of 90.2% for the QSAR4:1-R random model and 91.1% for the QSAR4:1 rational model. The overall coverage improvement effect of the rational selection depended on the prediction set: We saw no substantial improvement in the test set coverage but an increase in the overall coverage of the REACH set from 54.9% in the QSAR4:1-R random model to 63.1% for the QSAR4:1 rational model, i.e. an increase of 15% in the rational model compared to the random. However, from the external validation we saw that coverage of AhR activators was smaller for the rational QSAR4:1 model compared to the random QSAR4:1 model.

The Rational-final and Random-final models where the 93 test set actives and four times this number, i.e. 372, inactives were added to the training set showed similar or even better predictivity and robustness compared to their predecessors according to external validation for specificity and in the two times five-fold cross-validations. According to external validations with 154,513 experimental inactives, the Rational-final model has a specificity of 96.8% and the Random-final model has a specificity of 91.3%. Both models also showed better robustness than their predecessors, with the Random-final model showing the best robustness.

The top twenty most significant and discriminating predictive structural features associated with AhR activation actives and inactives, respectively, were identified in the Rational-final and Random-final models and may be used in further studies on mechanisms for AhR activation or possibly be taken into consideration in the design of future chemical substances.

The Rational-final and the Random-final models were applied to screen a set of 80,086 REACH substances. The Rational-final model predicted 1,256 substances (2.5% out of the 50,561 substances in AD) to be POS_IN versus the Random-final model, where 3,214 (7.1% out of the 45,557 substances in AD) were predicted POS_IN. The two final models show extremely good agreement (MCC of 0.983) in their common domain of the REACH set.

The final models as well as predictions for AhR activation for 650,000 substances will be published in the Danish (Q)SAR Database and can, for example, be used for priority setting, in read-across predictions and in IATA weight-of-evidence assessments of chemical substances. As the Rational-final model has higher specificity and the Random-final model has higher sensitivity and AhR activators coverage, we recommend using predictions from both models in concert.

## Abbreviations

AD: applicability domain
AhR: aryl hydrocarbon receptor
AOP: Adverse Outcome Pathway
CYP: cytochrome P450
CYP3A4: human cytochrome P450 isoform 3A4
DTU: Technical University of Denmark
FN: false negative
FP: false positive
HAH: halogenated aromatic hydrocarbons
HTS: high-throughput screenings
LPDM: Leadscope Predictive Data Miner
MCC: Matthews correlation coefficient
MIE: molecular initiating event
NEG_IN: a negative prediction in the applicability domain
NEG_OUT: a negative prediction out of the applicability domain
NPV: negative predictive value
PAH: polycyclic aromatic hydrocarbons
POS_IN: a positive prediction in the applicability domain
POS_OUT: a positive prediction out of the applicability domain
PPV: positive predictive value
*q*HTS: *quantitative* HTS
PXR: Pregnane X Receptor
QSAR: quantitative structure-activity relationship
TH: thyroid hormone
TN: true negative
TP: true positive
QSAR*m*:1: QSAR model with a ratio of *m*:1 between inactives and actives in the training set, *m* = 2, 3 or 4, REACH, Registration, Evaluation, Authorisation and restriction of Chemicals
